# Statistical validation of the criteria for symptom remission in schizophrenia: Preliminary findings

**DOI:** 10.1186/1471-244X-7-35

**Published:** 2007-07-24

**Authors:** Mark GA Opler, Lawrence H Yang, Sue Caleo, Philip Alberti

**Affiliations:** 1 Columbia University, Department of Psychiatry, New York, NY, USA; 2 Health Economics, Janssen Pharmaceutica N.V., Belgium; 3 Columbia University, Department of Epidemiology, New York, NY, USA; 4 The PANSS Institute, New York, NY USA

## Abstract

**Background:**

Published methods for assessing remission in schizophrenia are variable and none have been definitively validated or standardized. Andreasen et al (2005) suggest systematic operational criteria using eight PANSS items for which patients must score ≤ 3 (mild) for at least six months.

**Methods:**

Using data from a one year, multi-site clinical trial (n = 675) remission criteria were compared to total PANSS scores and other endpoints and demonstrate excellent agreement with overall clinical status.

**Results:**

Compared to total PANSS score of 60 points and other criteria, at time points > 6 months (8 and 12 months) the specificity of the remission criteria was 85%, i.e. of the patients who had a total score >60, 85% were classified as "not in remission." Sensitivity was also very high; 75% of patients with scores of <60 were classified as "in remission."Patients who dropped out of the trial were more likely not to be in remission prior to dropping out.

**Conclusion:**

These findings indicate that the remission criteria are both sensitive and specific indicators of clinical status. Additional analyses are required to determine if remission status predicts other outcomes, such as employment, independent living, and prognosis.

## Background

Prevention and recovery for patients with schizophrenia and other psychotic disorders are becoming the principle objectives for research and treatment. [1] While promising findings suggest that these goals will soon be attainable, efforts have also focused on development of interventions intended to reduce symptom severity over time. By maintaining low levels of psychopathology, such interventions may promote productive social and occupational pursuits and help contribute to the ultimate goal of recovery from illness. Consequently, researchers and clinicians have begun to seek consensus on the concept of symptom remission. Current methods for assessing remission vary from study to study, often utilizing measures of symptom severity developed for other purposes. In the past, remission in schizophrenia has been measured either by improvements in overall psychopathology from baseline (as measured by statistically significant percentage reductions in all thirty Positive and Negative Syndrome Scale (PANSS) items) or by reduction below specific threshold levels (e.g.: scores of less than 3, Mild in all thirty PANSS items). [2,3]

While it makes intuitive sense to utilize instruments that are well characterized in clinical research, none have been definitively validated as measures of remission. Additionally, when used according to current standards, some interview tools (e.g. the Structured Clinical Interview for the PANSS or SCI-PANSS) require extensive time to complete as they incorporate a number of questions and probes that may not be useful for assessing remission. The process of developing and testing a measure of remission based on threshold levels of selected PANSS items should be differentiated from previous efforts to create shortened versions of existing scales (e.g. the SF-12, derived from the SF-36). Rather than substitute for the 30-item PANSS, development of a concise outcome measure for remission in schizophrenia would create a benchmark for treatment and maintenance goals in clinical research and general practice. By developing an approach based on uniform criteria rather than the various criteria currently in use, a more meaningful standard for assessing remission status can be applied in both research and clinical practice.

### The Remission Construct

Early in 2005, Andreasen et al published an article in the American Journal of Psychiatry on strategies to define remission in schizophrenia through succinct operational criteria using threshold levels maintained over time. Through review of previous work and historically accepted constructs, Andreasen et al identified three primary areas of concern – Psychoticism, Disorganization, and Negative Symptoms (Psychomotor Poverty). These were then related to DSM criteria and finally to specific items from various psychometric instruments. Eight PANSS items (see Table 1) based on this framework were identified. The authors suggested that in order to qualify for remission status, patients must score less than or equal to 3 (mild) on these eight items. This must be maintained for at least six months. Remission was also described as "indicating progress towards recovery" associated with sufficiently low levels of psychopathology such that behavior is no longer markedly affected. A number of publications have come out in support of this construct (van Os et al 2006), including an important report by Docherty et al. (2007) showing that the remission criteria was associated with low CGI scores and improvements in patient reported outcomes.[4,5]

**Table 1:** PANSS Items for use in Symptoms of Remission

**Name**	**Item #**	**Sources of Information**
Delusions	P1	Interview & Informant Report
Unusual thought content	G9	Interview Only
Hallucinatory behavior	P3	Interview & Informant Report
Conceptual disorganization	P2	Interview Only
Mannerisms/Posturing	G5	Interview & Informant Report
Blunted affect	N1	Interview Only
Passive social withdrawal	N4	Informant Report Only
Lack of spontaneity/Flow of Conversation	N6	Interview Only

PANSS Remission items and sources of information.

### Symptomatic and Functional Remission

Weiden et al. suggest that functional endpoints are not independent from psychopathology, describing symptomatic "stabilization" as a prerequisite for improvements in cognition, quality of life, and related areas. [6] Supporting evidence from the literature shows that specific aspects of psychopathology seem to be related to occupational function. Some reviews of the subject have critiqued psychometric rating scales for their failure to incorporate functional outcomes. This is a valid concern and one that requires additional attention and development. However, several instruments do take the impact of symptoms on function into account, such as the PANSS. While most of the PANSS items that are included in Table 1 are made largely on the basis of clinical observation, three are also based in part on functional impact over the past week. A fourth item (Passive/apathetic social withdrawal) is based solely on informant report of impairments in social function. Although these items may not provide a thorough indication of functional status, if utilized correctly, they should provide some evidence of the impact of disease severity on progress towards functional remission.

### Psychometric Properties of PANSS Remission Items

In an effort to determine how to group PANSS items in meaningful ways beyond the conceptually derived Positive, Negative, and General Psychopathology subscales, several rigorous factor analytic approaches have been applied to large PANSS datasets. PANSS remission items may be considered according to their factor loadings. Using the PANSS "Pentagonal Model" loadings of White et al (1997) and the six-factor solution of van den Oord et al. (2005) the eight Remission items account for the highest loading items in the Positive, Negative, and Disorganized factors (P1 and G9, N6 and N1, respectively) as well as a number of the additional high to moderately loading items in the Withdrawal, and Autistic Preoccupation factors (see Table 2). [7,8]

**Table 2:** Psychometric Properties of PANSS Remission Items

**Item #**	**Sources of Information**
P1	Highest loading item for Pentagonal (0.89) and Hexagonal Positive Factor (0.81)
G9	2^nd ^highest loading item for Pentagonal (0.79) and Hexagonal Positive Factor (0.68)
P3	Primary loading on Pentagonal Positive Factor (0.43) and Hexagonal Positive Factor (0.28) Secondary on Pentagonal Autistic Preoccupation Factor (0.31)
N6	Highest loading item for Pentagonal (0.84) and Hexagonal Negative Factor (0.75)
N1	2^nd ^highest loading item for Pentagonal Negative Factor (0.78) and Hexagonal Positive Factor (0.65)
N4	High loading on Pentagonal Negative Factor (0.689) and Hexagonal Withdrawal Factor (0.89)
G5	Moderate loading on Pentagonal Negative Factor (0.44) and Hexagonal Disorganized Factor (0.28)
P2	Highest loading on Pentagonal (0.74) and Hexagonal Disorganized Factor (0.75)

Loadings for PANSS remission items in factor-analysis derived models

Recently, researchers have conducted retrospective analyses using these new remission criteria to determine whether remission may be used as a study outcome. Lasser et al (2005), as well as Sethuraman et al (2005) and others have used the eight PANSS items selected by Andreasen et al to identify remitted patients within randomized clinical trials and characterize symptom severity and other features.[9] Results show that remitted patients had lower total PANSS scores, spent fewer days as inpatients, had shorter duration of illness, and used fewer prescription psychotropics.

The studies published to date have provided valuable insight into the promise of objective, standardized approaches to assessment of remission status. However, there are several outstanding questions that cannot be readily addressed without additional work. The first question is whether any consensus measure reliably identifies patients who have low levels of psychopathology. Standard statistical tests, such as sensitivity and specificity, agreement over chance, and positive predictive value must be conducted. The second question is whether the longitudinal criteria recommended by Andreasen and colleagues (i.e. maintaining low levels of psychopathology for 6 months or more) improves the validity of the measure.

## Methods

The PANSS is a thirty item scale, with individual item scores from 1–7. Total scores range from 30–210. Using PANSS data from a prospective, multi-site trial, symptom severity was compared in patients in remission vs. those not in remission as defined by the operational criteria described above (scores ≤ 3 for all eight remission items). Based on work by Leucht and others, two sets of cutoffs, one for a "mildly ill" total PANSS Score (≤ 60) and a moderately low total PANSS Score (≤ 75) were used as the "gold-standards" in this analysis. [10] The analysis was first conducted in cross-sectional fashion at each visit, comparing total PANSS scores in subjects who meet symptom severity criteria for remission vs. non-remitted subjects.

Data from a previously published randomized clinical trial (ROSE Study, described below) was analyzed longitudinally through 4-month, 8-month, and one-year time points in subjects who meet symptom severity and timeframe criteria for remission vs. non-remitted subjects. In this analysis, remission criteria were compared in both cross-sectional fashion (determining remission status at a single time point based solely on symptom severity) and also using the longitudinal requirements, i.e. defining remission based on prior status over time. For example, remission status at 8 months was established using data from 4- and 8-month visits and remission at 1 year was established using data from 4 months through 8-months for the same subjects. Using the described threshold for symptom severity levels with further restriction to those who also meet longitudinal requirements, subjects were grouped according to the classifications shown in Table 3. This allowed both cross-sectional and longitudinal approaches to be compared.

**Table 3:** Classification for Measures of Agreement

	***Low/Moderate *(≤ 75)*, Low *(≤ 60) *Symptom Severity***	***High Symptom Severity***	***Totals***
***In Remission***	a. In remission, low/moderate, low symptom severity	b. In remission, high symptom severity	a + b. Total in Remission
***Not In Remission***	c. Not in remission, low/moderate, low symptom severity	d. Not in remission, high symptom severity	c +d. Total Not in Remission
***Totals***	a + c. Total Low/Moderate Low Symptom Severity	b + d. Total High Symptom Severity	Combined Totals

Remission vs. Symptom Severity Definitions.

Based on the cells identified in Table 3, analyses of four parameters were calculated: sensitivity, specificity, positive predictive value (PPV) and negative predictive value. Logistic regression techniques were also be used to calculate likelihood of remission status over time (with past remission status used to predict remission status at a specific time point).

### The ROSE Study

This analysis is based on data collected from the ROSE Study, a one-year multi-center, open-label, randomized clinical trial carried out in 21 centers in 17 US states (Mahmoud et al, 1999). Patients were randomly assigned to treatment with either risperidone or a conventional antipsychotic drug (n = 684). Follow-up was conducted from 1995–1997. [11]

Inclusion criteria for the study included patients aged 18–60 y.o., diagnosed with schizophrenia or schizoaffective disorder according to ICD-9-CM criteria. For inclusion, diagnosis had to be made prior to age 35 with at least one hospitalization in a locked facility in the 2-years prior to the study, with symptom relapse at study start as defined by either (a) an inpatient admission with a mental disorder within 10 days of study start-up or (b) an emergency room visit, contact with crisis psychiatric treatment services, or (c) an unscheduled office or clinic visit accompanied by exacerbation of symptoms of schizophrenia. The PANSS was administered every 4 months, i.e. at baseline (T1), at 4 months (T2), at 8 months (T3), and at 12 months (T4).

### Demographics

A majority (69%) of the patients in this study were male. Average age was 38.5 y.o. (SD = 9), with 59.7% of subjects identified as White, 26.5% Black, 4.7% Hispanic, 4.6% Asian, and 4.4% in other categories. Mean age of onset of psychiatric symptoms was 22 y.o. (SD = 6), making the mean course of illness 16 years by the start of the study. The majority of subjects had never been married at the time the study was conducted (58.8%) and were inpatients (65.5%).

## Results

A correlation matrix between the 8-item remission scale and total PANSS scores revealed negative and significant relationships at the p < .001 level at all four time points. Oneway ANOVA procedures demonstrate that the remission subscale scores explained a significant amount of the variance in positive, negative, general and total PANSS scores at T2 (four months), T3 (eight months) and T4 (one year), although not at T1 (baseline). Scores were significantly lower for remitted subjects at all each of these three follow-up visits (see Table 4). All between-group differences were statistically significant (p < 0.001(.

**Table 4:** Total PANSS Scores for Patients by Remission Status, T2-T4

		N	Mean	Std. Deviation	Std. Error
4 Month Visit (T2)	Not In Remission	398	81.02	17.47	.875
	In Remission	162	55.81	11.65	.915
	Total	560	73.73	19.66	.831
8 Month Visit (T3)	Not In Remission	335	76.77	16.95	.926
	In Remission	180	53.57	10.92	.814
	Total	515	68.66	18.73	.825
12 Month Visit (T4)	Not In Remission	261	75.06	17.13	1.060
	In Remission	166	51.77	11.19	.869
	Total	427	66.01	18.89	.914

Mean total scores for study subjects, grouped according to remission status from T2-T4.

At T1, when all subjects were in relapse according to ROSE study definitions as described above, the 8-item criteria correctly identified almost all (647 of 667) as not in remission. If 'disease' is classified as study-defined relapse and compared to the 8-item remission measure, then sensitivity = .97 (647/667), specificity = 1.00 (0/0), positive predictive value = 1.00 (647/647), and negative predictive value = 0 (0/20) The PANSS total score was used to define two cutoffs of 60 and 75 (see Tables 5 &6). For T2-T4, 70–73% of those with a total PANSS score of less than 60 met the remission criteria as compared with 51–52% of subjects with total scores of less than 75. At each successive visit from T2-T4, the positive predictive value of the remission criteria vs. a total PANSS score < 60 increases by approximately 10%, going from 60% at T2, to 70% at T3 and 80% at T4 while remaining consistently high (95%–98%) as compared to a total PANSS score < 75.

**Table 5:** 8-Item Remission Criteria vs. Total PANSS Score < 60

Visit	n	Sensitivity	Specificity	PPV
T1	636	.27	.99	.60
T2	560	.72	.85	.61
T3	515	.70	.83	.69
T4	427	.73	.86	.79
Mean	535	.68	.90	.69

Sensitivity, specificity, and PPV of the Remission criteria as compared to a total score <60.

**Table 6:** 8-Item Remission Criteria vs. PANSS < 75

Visit	n	Sensitivity	Specificity	PPV
T1	636	.09	.995	.90
T2	560	.51	.97	.95
T3	515	.51	.98	.98
T4	427	.52	.95	.96
Mean	535	.44	.98	.96

Sensitivity, specificity, and PPV of the Remission criteria as compared to a total score <75.

### Longitudinal Findings

Although positive correlations were seen when T1 remission scores were compared to T2, T3 or T4, these associations were not statistically significant. However, all associations between T2, T3 and T4 scores were significantly correlated (p < .001; r's ranged from 0.39-.4.8). When measured across all four timepoints, correlations between the Positive subscale (0.3–0.65), Negative subscale (0.41–0.66), and General Psychopathology subscale (0.30–0.60) were significantly correlated (p < 0.001).

### Predicting Remission Status

Logistic regression models were fit in order to calculate the likelihood of maintaining remission based on status at prior visits (see Table 7). Status at two consecutive visits was used to predict the status at the third visit. For subjects with complete PANSS data at T3 (N = 515), being in remission at both T1 and T2 demonstrated a trend towards prediction of remission at T3 (p = .064) with an Odds Ratio = 4.8 (95% Confidence Interval: 0.91–24.8) For subjects with complete PANSS data at T4 (N = 427), being in remission at both T2 and T3 was a highly significant predictor of remission at T4 (p < 0.001) OR = 9.7 (95% Confidence Interval: 5.4–17.4).

**Table 7:** Predicting Remission Status

If in Remission at Visit:	Predicted Remission Status at Visit:	n*	Odds Ratio	95% Confidence Interval	p-value
T1+T2	T3	515	4.8	0.91–24.8	0.064
T2+T3	T4	427	9.7	5.4–17.4	<0.001

*Indicates number of subjects with complete PANSS data at all relevant visits.

Estimated odds ratio for remission at T3 and T4 given status over prior 8 months.

### Dropouts

Analysis of subjects who dropped out of the study at T2-T4 demonstrated a tendency not to be in remission at one or more visits. Of the 71 dropouts at T3, 38 were not in remission at T2, and only 13 were in remission at T2. A Chi-Square test of this likelihood was significant at the p < 0.001 level. The estimated Odds Ratio for dropping out of the study given failure to achieve remission at the prior visit is 7.3 for T3 (95% Confidence Interval: 4.7–11.2) and 8.8 at T4 for subjects not in remission at prior visit (95% Confidence Interval: 5.6–13.9). (See Table 8.)

**Table 8:** Predicting Dropout Status

If not in Remission at Visit:	Predicted Droput Status at Visit:	n*	Odds Ratio	95% Confidence Interval	p-value
T2	T3	515	7.3	4.75–11.17	<0.001
T3	T4	427	8.8	5.56–13.91	<0.001

*Indicates number of subjects with complete PANSS data at earlier visit.

Estimated odds ratio for dropping out of the study at T3 and T4 given status at prior visit.

## Discussion

While a consensus is developing on one definition of symptom remission, it is important to not confuse this operational definition with the broader concept of recovery. As described by Resnick and others, recovery is multidimensional; reduction of psychotic symptoms represents just one aspect of recovery. This difference is clearly articulated by in studies that use both objective clinical data, as well as subjective endpoints such as patient self-experience and measures of hopefulness. Work by Lysaker and colleagues demonstrates that the objective and subjective elements of recovery are related, but (Lysaker et al., 2006) Our analysis was intended to test the statistical properties of one operational definition, based on previously published criteria.

Based on our findings, the remission criteria of Andreasen et al appear to be potentially valuable for future studies. First, these criteria demonstrate high specificity, sensitivity, and positive predictive value when compared to PANSS total score. The results of cross-sectional comparisons of two cut-offs of the total PANSS score (60 and 75) clearly show almost all subjects identified as being in remission at a given time point have low/low-moderate levels of psychopathology. At first glance, the results of this analysis at T1 (baseline visit) suggest that the remission criteria are not valid. However, it should be noted that very few subjects met the remission criteria at baseline (n = 30), making it difficult to draw any meaningful conclusions regarding total PANSS scores. By contrast, when study-defined relapse is examined, the remission criteria appear to have excellent predictive value.

Next, when longitudinal components of the criteria are included, the predictive value of the criteria improves. Specifically, using data from T2-T4, for subjects in remission at 4 months and 8 months, the likelihood of being in remission at 12 months is increased 10-fold versus those subjects who fail to be in remission at one or both of the preceding time points. The analysis of data from T1-T3 is problematic since exceptionally few subjects met criteria at T1, as noted previously. This may account in part for the trend towards significance and the wide confidence interval. The differences in the value of the remission criteria at T3 to T4 are only observable when available data from previous visits are included.

An interesting, if unexpected finding was the strong relationship between failure to achieve remission and risk of dropping out of the study. Similar results have been reported previously; Hatta et al. suggest that "treatment resistance" may be predicted using PANSS baseline scores on selected items, while other studies relate medication non-compliance to PANSS-measured mood symptoms and PANSS total score. [11-13] Through quantitative approaches to treatment (i.e. "targeted treatment" – see Opler et al., 2006 for a review) such findings are gradually being applied in various clinical settings. In future research, efforts might be made to study the reasons for medication discontinuation and correlations with psychopathology. [14]

### Limitations

One of the major criticisms of analyses based on samples drawn from randomized clinical trials is that they may not resemble the general population. Certain types of subjects are potentially excluded or under-represented (e.g. the elderly, those with co-morbid conditions, such as drug dependence). [15] In order to test the validity of the remission construct, particularly the extent to which it is generalizable to all patients with schizophrenia spectrum disorders, samples drawn from multiple populations should be compared.

The primary limitation of this analysis is that it does not incorporate an independent measure of functional status. While there is some data showing that changes in PANSS scores are related to quality of life measures, determining the best approach towards quantifying remission will require that both psychopathology and function be taken into account. [16] Although not generally acknowledged, the PANSS and similar scales are intended to incorporate both observed and reported impact of symptoms on function. In patients suffering from acute psychosis, the lack of insight necessitates the use of 'informants', or third-parties who can contribute data on the patient's behavior and activities over the past week. (Note: The clinician performing the interview is permitted to act as the informant if he or she has specific knowledge about the patient over the past week.) [17] While the conventions of the PANSS require that informant data be obtained and incorporated, it is rarely considered separately from the scale itself. In future research, informant data might be analyzed separately and jointly in the interest of determining whether a meaningful "functional impact factor" could be extracted from PANSS data, compared to independent, previously validated measures and potentially applied to new studies of remission in schizophrenia.

While the eight items included in this construct are positively associated with selected outcomes and have several useful properties, it is necessary to take an empirical approach to this question as well, i.e. utilizing statistical techniques to objectively determine which PANSS items are most strongly associated with remission over time. One possibility is the application of Item Response Theory to determine which PANSS items are most valuable for differentiating remitted and non-remitted patients; such approaches have been taken with depression rating scales. [19]

A practical concern is the need for standardized, efficient methods for data collection.

## Conclusion

In conclusion, our findings suggest that the operational criteria outlined by Andreasen and colleagues have potential for use in the future. However, we also conclude that our results are limited in several respects and key endpoints must be incorporated in future studies. Going forward, we intend to compare multiple operational criteria objectively, using rigorous techniques. In doing so, symptom remission should be considered as one aspect of the broader construct of recovery from illness – while future studies may incorporate symptom remission as one aspect of recovery (or possibly as one milestone in the process of recovery) the field must continue to strive for the best possible outcomes for our patients. Given the urgent need to continue to improve on "real-world" efficacy of antipsychotic treatment [20], no construct should be considered sacred and expectations should not be lowered based on limited data.

## Competing interests

Financial support for this study provided by Janssen Pharmaceutica N.V. Belgium. One author (S.C.) is employed by Janssen, none of the other authors have any potential competing interests.

## Authors' contributions

MGAO led the conceptualization and design of the study and drafted and edited the manuscript. PA carried out the statistical analyses and drafted tables and corresponding sections of the manuscript. LHY participated in the design of the study, reviewed and edited the manuscript. SC participated in the conceptualization and design of the study, reviewed and edited the manuscript. All authors read and approved the final manuscript.

## Pre-publication history

The pre-publication history for this paper can be accessed here:


